# Obesity, Physical Activity and Occurrence of High Medical Expenditures at One-Year Follow-Up Among Japanese Beneficiaries of Employment-Based Health Insurances: An Analysis Based on a Nationwide Health Checkup Questionnaire

**DOI:** 10.3390/healthcare14060777

**Published:** 2026-03-19

**Authors:** Aya Higashiyama, Yuki Yonekura, Nagako Okuda, Kozo Tanno, Akira Okayama

**Affiliations:** 1 Department of Hygiene, Wakayama Medical University, 811-1 Kimiidera, Wakayama-Shi 641-8509, Japan; 2 Department of Nursing Informatics, Graduate School of Nursing Science, St. Luke’s International University, 10-1 Akashi-Cho, Chuou-Ku, Tokyo 104-0044, Japan; yyonekura@slcn.ac.jp; 3 Division of Applied Life Sciences, Graduate School of Life and Environmental Sciences, Kyoto Prefectural University, 1-5 Sshimogamohanki-Cho, Kyoto-Shi 606-8522, Japan; nokuda@kpu.ac.jp; 4 Department of Hygiene and Preventive Medicine, Iwate Medical University, 1-1-1 Idaidouri, Yahaba-Cho 028-3694, Japan; ktanno@iwate-med.ac.jp; 5 The Research Institute of Strategy for Prevention, 10-14 Nihonbashi Tomizawa-Cho, Chuou-Ku, Tokyo 103-0006, Japan; aokayama@jrisp.com

**Keywords:** overweight, exercise, body mass index, abdominal obesity, health insurer, employees’ health insurance

## Abstract

**Objectives:** This study aimed to prospectively investigate the associations among obesity, physical activity, and short-term high medical expenditures in Japanese employees and their dependents. **Methods:** Participants were 245,044 employees and their dependents aged 40–74 years who underwent the Specific Health Checkup in fiscal year 2008. Health checkup and medical expenditure data for 2008–2010 were provided by health insurers. They were divided into 12 groups according to the combination of body mass index categories (normal, overweight, and obesity) and engagement in exercise and/or daily physical activity (inactive, daily physical activity only, exercise only, and active). The multivariable-adjusted odds ratios of the groups for high total medical expenditures in the next year compared to the inactive normal body mass index group were estimated. High medical expenditures were defined as the top 5% of total annual expenditures, consistent with prior literature identifying high-cost users. Similar analyses were performed by sex and age (<65 years, ≥65 years). **Results:** Of the participants, 61.8% were men (mean age, 52.1 years). Multivariable-adjusted odds ratios were significantly high only in the inactive groups with overweight or obesity in men and younger individuals. In women and older individuals, the odds ratios were significantly high only in inactive women with obesity; however, the odds ratios were high in women who exercised only and in active older individuals, both with obesity. **Conclusions:** Exercise or daily physical activity might attenuate the possibility of incurring high short-term medical expenditures in men and younger individuals with overweight or obesity. These findings suggest that physical activity recommendations may need to be tailored for women and older individuals with obesity, and further longitudinal research is warranted.

## 1. Introduction

Obesity (OB) is associated with multiple complications including type 2 diabetes, cardiovascular disease (CVD), cancer, metabolic dysfunction-associated fatty liver disease, respiratory disorders, and musculoskeletal disorders, leading to increased morbidity and medical expenditures (ME) [1]. In Japan, all citizens are covered under the universal health insurance. Workplaces legally mandated by this system must provide Employees’ Health Insurance (EHI), enrolling regular employees, officers, and representatives. The EHI covers medical care and related allowances for insured individuals and their dependents, excluding work-related injuries and occupational accidents. Insurers are responsible for most ME and implementing health services, including Specific Health Checkups (SHC) and Health Guidance (SHG) which aim to reduce the risk of lifestyle-related diseases and future ME among all Japanese residents aged 40–74 years by decreasing the number of individuals with OB, such as visceral fat-type OB [2]. Therefore, effective OB management is critical for the health of insured members and the sustainable operation of health insurers.

Physical activity (PA), including exercise and routine daily activities, such as walking, is a recommended lifestyle intervention for health promotion [3]. Previous studies have suggested that higher PA is associated with lower ME [4,5,6] and that the combination of OB and lifestyle factors, including PA, may influence short-term ME, as reported in a clinic-based Western population [7]. However, few studies have investigated the combined effect of OB and different types of PA on short-term high ME in large occupational cohorts, which are generally healthier than the general community-based population [8].

Recently, pharmacological treatments for patients with OB, including those with a body mass index (BMI) below 30 kg/m^2^ and OB-related comorbidities, have become one of the therapeutic options in Japan [9]. The increasing availability of anti-OB medications may alter the long-term relationship between lifestyle factors and ME, making pre-pharmacotherapy baseline evidence particularly valuable.

Using data collected in fiscal year (FY) 2008–2009, the present study prospectively investigated the association between OB-related anthropometric indices, PA assessed by the questionnaire in the SHC, and high ME (top 5% of ME in the participants) one year later among approximately 240,000 Japanese beneficiaries of the EHI, most of whom were employees.

## 2. Materials and Methods

### 2.1. Participants

**Figure 1:**
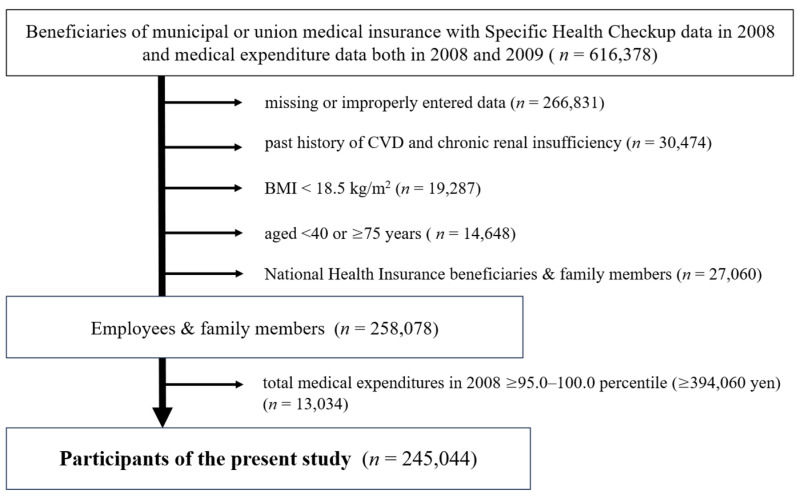
Flow chart of the selection for determining the participants.

Participants were drawn from a Health and Labour Sciences Research, The Influence of Specific Health Checkups and Specific Health Guidance by Medical Insurers on Medical Expenditures (Grant-in-Aid for Health and Labour Sciences Research, H20-Policy, General-014, FY 2008–2010, principal investigator: Akira Okayama) [10], and the present study was conducted as a secondary analysis of data from the above-mentioned research project. In this project, data on insured individuals’ ME and SHC during the study period (FY 2008 and 2009 (April 2008–March 2010)) were obtained from six insurers providing EHI and 12 municipal insurers providing National Health Insurance (NHI). Among 616,378 individuals (563,111 individuals from EHI and 53,267 from NHI), participants with missing data on BMI, PA or covariates were excluded (*n* = 266,831). The proportion of missing data for each variable ranged from 0.001% to 35.4%. In addition, participants those who were aged <40 years or ≥75 years, and with past history of CVD (stroke and/or heart disease) and/or chronic renal insufficiency, BMI < 18.5 kg/m^2^ were excluded. Among the remaining 285,138 individuals (258,078 EHI beneficiaries and 27,060 NHI beneficiaries), EHI beneficiaries were included for the following reasons: (1) the distributions of ME during the data collection period were different between EHI and NHI beneficiaries, as more elderly individuals were covered by NHI, and ME in NHI beneficiaries were distributed toward the upper end of the distribution compared with those among EHI beneficiaries; and (2) EHI beneficiaries were generally younger and healthier [8] and they represented a suitable population for evaluating preventive approaches by lifestyle modification aimed at mitigating future high ME. Because the outcome of this study was high ME (95.0–100.0 percentile in the participants [11]) in 2009, we further excluded EHI beneficiaries whose total ME in 2008 (baseline year, ME2008) was high (95.0–100.0 percentile among 258,078 EHI beneficiaries) from the study population. The remaining 245,044 beneficiaries were included in the analysis (Figure 1).

### 2.2. Anthropometric and Lifestyle Measures

Anthropometric measurements and demographic variables, including age and sex, were obtained from the SHC results in 2008. BMI was classified as normal weight (NW) (≥18.5 and <25.0 kg/m^2^), overweight (OW) (≥25.0 and <30.0 kg/m^2^), or OB (≥30 kg/m^2^). Abdominal obesity (AO) was defined as waist circumference (WC) ≥ 85 cm for men and ≥90 cm for women. Hypertension was defined as systolic blood pressure (SBP) ≥ 140 mmHg and/or diastolic blood pressure (DBP) ≥ 90 mmHg and/or taking antihypertensive medication. Diabetes was defined as a fasting blood glucose level ≥ 126 mg/dL, a casual blood glucose level ≥ 200 mg/dL, or the use of antidiabetic medication. Dyslipidemia was defined as a low-density lipoprotein cholesterol (LDL-C) level ≥ 140 mg/dL [12] or taking lipid-lowering medication.

PA and other lifestyle factors in 2008 were assessed using responses to the standard SHC 2008 questionnaire. Participants who answered “yes” to the question, “Are you in a habit of doing exercise to sweat lightly for over 30 min a time, 2 times weekly, for over a year?” were classified as exercising (Ex)+. Participants who answered “yes” to another question, “In your daily life do you walk or do any equivalent amount of PA more than one hour a day?” were classified as having daily PA (dPA)+. Participants’ PA status was classified into four groups based on the combination of participation in Ex and dPA: Inactive (dPA−/Ex−), dPA (dPA+/Ex−), Ex (dPA−/Ex+), and Active (dPA+/Ex+). Current smoking was defined as habitual smoking at the time of the SHC, and alcohol consumption was categorized into three groups: daily drinkers, occasional drinkers, and rare drinkers, including never drinkers.

### 2.3. Statistical Analysis

Baseline characteristics were compared among the 12 groups classified by the combination of the four PA categories (Inactive, dPA, Ex, and Active) and the three BMI categories (NW, OW, and OB) by sex.

The 95th percentile threshold (95.0–100.0 percentile among the participants) was selected to identify high ME users, consistent with previous health economics research, in which high-cost users were commonly defined as those in the top 5% of healthcare expenditures [11]. The proportions of the participants with high ME and zero ME in next year (HME2009 and noME2009) were calculated according to the BMI categories. The odds ratios (ORs) and 95% confidence intervals (95% CI) of OW or OB for HME2009 compared with NW (reference) were estimated after adjusting for confounders in all participants. Model 1 was adjusted for age, sex, hypertension, diabetes, dyslipidemia, smoking, and alcohol consumption to control for demographic characteristics and baseline health conditions that may influence ME. In Model 2, baseline ME (ME2008) was additionally included to account for pre-existing differences in healthcare utilization and initial costs, to investigate whether BMI and PA were associated with HME regardless of baseline ME.

The proportion of participants with HME2009 or noME2009 was calculated for the 12 groups classified according to the PA and BMI categories. The multivariable-adjusted ORs and 95% CI for HME2009 in the 11 groups were estimated after adjusting for the same confounders using the Inactive and NW group as the reference in all participants. Using the logistic regression model, we calculated predicted probabilities of each BMI–PA groups for HME2009, after adjusting for the same confounders in Model 2. The same analyses as described above were performed after stratification by sex and by age groups (≤64 years and 65–74 years). Those sex and age group-specific analyses were conducted as pre-specified subgroup analyses. In addition, the same analyses were performed using two categories of AO instead of the BMI categories. Because 45 participants had no WC data, the analysis using AO categories was performed on 244,999 participants.

To examine the impact of raising the threshold for high ME, sensitivity analyses were conducted using two additional definitions of high ME as outcomes—(1) the 96.0–100.0 percentile of all participants in 2009 (HME2009_2), and (2) expenditures of Japanese yen (JPY) 1,000,000 or more in 2009 (HME2009_3)—in which the multivariable-adjusted ORs in Model 2 were estimated in the 11 groups defined by the combinations of the BMI and PA categories, using the Inactive and NW group as the reference. In the analyses with HME2009_2 and HME2009_3 as outcomes, participants who met the corresponding definitions in the baseline year were excluded.

All statistical analyses were performed using SPSS version 26 (IBM Japan Ltd., Tokyo, Japan).

## 3. Results

Among the study population, 61.8% were men, 95.4% were aged 40–64 years, and the mean age was 52.1 ± 7.4 years. The proportion of employee dependents was 0.4% and 28.9% for men and women, respectively. The prevalences of OW and OB were 23.6% and 3.6%, respectively. Regarding PA, 61.9%, 18.7%, 8.1%, and 11.3% of the participants were Inactive, dPA, Ex, and Active, respectively. In FY2009, 22.4% of the study population was noME2009 and the maximum ME was JPY20,361,990. The cutoff for the HME2009 was JPY336,380 (Table 1).

Baseline characteristics of the participants are presented in Table 2 (men) and Table 3 (women). Across all PA categories, the prevalence of hypertension, diabetes, and dyslipidemia as well as the median ME2008 were generally higher in higher BMI categories in both men and women, whereas the prevalence of daily alcohol consumption tended to be higher in lower BMI categories. Age showed different patterns by sex, tending to be higher in lower BMI categories in men and highest in the OW group in women.

The proportions of participants with HME2009 or noME2009 and the ORs of the BMI categories for HME2009 in all participants are presented in Table 4.

The proportion of participants with HME2009 was higher in the higher BMI category, whereas that with noME2009 was lower. In Model 2, the ORs of OW and OB were significantly higher than those of NW in all participants. In sex-specific (Table 5) and age group-specific (Table 6) analyses, most of the results were similar to those in all participants; however, in older participants, the OR of OB in Model 1 and ORs of OW and OB in Model 2 were not statistically significant.

The proportions of participants with HME2009 or noME2009 and the ORs of the 11 groups classified by PA and BMI categories for HME2009 compared to Inactive with NW in all participants are presented in Table 4. In all PA categories, the proportion of HME2009 was higher in the higher BMI categories, whereas the proportion of noME2009 was lower. The sex- and age-adjusted ORs were significantly higher, except in the NW groups. In Model 1, the significant association disappeared in dPA with OW. In Model 2, significantly higher ORs were observed only in Inactive with OW or OB. In addition, a significantly lower OR of dPA with NW was observed in all models. Overall, higher BMI categories tended to be associated with higher odds of HME2009, whereas engagement in PA attenuated this association, with significantly higher ORs remaining only among Inactive with OW or OB.

The results of sex-specific analyses corresponding to those in Table 4 are shown in Table 5. Among women, in the dPA group, the proportion of noME2009 was the lowest in OW, while in the Active group, the proportion of HME2009 was the highest and that of noME2009 was the lowest in OW. In women, Model 2 showed significantly higher OR of HME2009 in Inactive with OB, and a significantly lower OR in dPA with NW. In addition, the OR of the Ex with OB group was higher, without statistical significance, compared to the other groups.

The results stratified by age group are shown in Table 6, corresponding to those in Table 4. Among older individuals, in the dPA group, the proportion of HME2009 was the highest in the OW category. Among younger participants, the ORs were similar to those observed for all participants. Among older participants, no significant association was observed in Model 2; however, the OR of the Active with OB group was higher compared with the other groups.

The predicted probabilities for each BMI–PA group ranged from 2% in dPA with OB to 13% in Active with OB, both for older individuals (Table 4, Table 5 and Table 6). The probabilities tended to be high among older participants and showed a similar pattern to the ORs in the overall population and across all subgroups.

The results of the analysis using AO instead of BMI-based categories are presented in the Supplementary Materials (Tables S1–S3). These results were almost similar to those obtained using BMI categories.

The results of the Model 2 analyses, with the outcome changed to HME2009_2 or HME2009_3, along with those for the original outcome (HME2009), are shown in Supplementary Materials (Figure S1). Among all participants, as well as in each subgroup, the tendency for a higher OR of Inactive with OB remained, even with a higher cutoff for HME. For HME2009_2, the OR of OB became lower from Inactive to Active among all participants, men, and younger individuals. For HME2009_3, the OR was significantly lower in Active men with NW. In addition, among women, the OR of Ex with OB was higher at a higher cutoff for HME, with statistical significance for HME2009_3. In older participants, the OR of Active with OB for HME2009_3 was not high, whereas the ORs were high for HME2009 and HME2009_2. In addition, in all participants, the ORs for HME2009 were statistically significant—higher in Inactive with OW and lower in dPA with NW—however, this significance was not observed for HME2009_2 and HME2009_3.

We additionally conducted the following sensitivity analyses among all participants, using the same model as in Table 4. In an analysis restricted to participants with zero ME in 2008 (noME2008), the overall pattern of results was not substantially different from those shown in Table 4. Furthermore, in an analysis restricted to participants with noME2008 who had none of the following conditions at baseline—hypertension, diabetes, or dyslipidemia—a significantly high OR was observed only in the Ex with OB (OR ≈ 4). This finding may suggest that a portion of the high ME observed among individuals with OB may not be directly related to conditions such as hypertension, diabetes, or dyslipidemia, but could instead be attributable to other factors related to PA. Because a sufficient number of events could not be secured in these additional analyses, stratified analyses by sex and age group were not conducted.

## 4. Discussion

In this large cohort of approximately 240,000 Japanese individuals, most of whom were male employees under 65 years of age, we found that only Inactive with OW or OB were significantly associated with high ME at 1 year. These findings suggest that engaging in any form of PA may be associated with lower short-term ME, even among individuals with OW or OB. These tendencies were particularly strong in men and younger individuals. Furthermore, although there was no statistically significant difference, the point estimate of the OR was high for the Ex with OB group in women and the Active with OB group in older individuals. Increasing the cutoff for high ME increased these trends in women with OB who engaged in exercise. This finding suggests that caution may be warranted when promoting exercise in this subgroup.

Few studies have prospectively examined the association between high ME and the combined categories of BMI and PA in a large employed population. In a previous clinic-based study in the United States, annual ME was estimated over a 1.5-year follow-up period for groups classified by combinations of BMI categories, engagement in PA more than three days per week, and current smoking. In this previous study, never smokers with PA and a BMI < 25.0 kg/m^2^ had a mean ME that was 49% lower than that of inactive current smokers with a BMI of 27.5 kg/m^2^ [7]. To the best of our knowledge, the present study is the first to prospectively investigate the association between ME and a combination of OB-related indices and PA in a large cohort consisting mainly of employees.

In Japan, where efforts are underway to reduce the number of individuals incurring high ME through OB prevention using SHC and SHG, it is important to examine the associations among OB-related indices, lifestyles associated with OB prevention, and ME in working individuals, who constitute the majority of the Japanese population. A major strength of this study is its use of a simple, standardized, and nationally unified questionnaire to assess PA, combined with a large sample size. We demonstrated an association between OB, PA, and future short-term ME in a predominantly working-age Japanese population characterized by a lower prevalence of OB than Western populations [13]. However, the findings may not be directly generalizable to the unemployed, informal workers, older community-based populations, or populations with higher baseline morbidity.

Despite these strengths, this study is a secondary analysis of data collected over a limited period for the Ministry of Health, Labour and Welfare research project. Consequently, the follow-up period was short and many individuals had zero ME in the subsequent year, making it difficult to investigate absolute ME values. The recent availability of large-scale real-world datasets linking SHC with individual ME allows analysis over extended follow-up periods. Therefore, an investigation of the long-term effects of OB-related indices and PA on ME is feasible and warranted.

In this study, individuals with OB had higher odds of high ME at 1 year compared to those with NW, while those with AO had higher odds compared to individuals with normal WC. All associations were statistically significant. A previous community-based study in the U.S. also reported that after excluding underweight individuals, a higher BMI was associated with increased ME. In this previous study, the lowest expenditures occurred at a BMI of 20.5 kg/m^2^ in women and 23.5 kg/m^2^ in men [14], corresponding to the normal BMI in the present study for both sexes, indicating a trend similar to our findings.

Several international cohort studies with relatively long follow-up periods have re- ported that OB and PA are associated with increased healthcare utilization and ME [15,16,17]. However, these studies have largely been conducted in Western populations, where the prevalence of OB is substantially higher than in Japan. Meanwhile, previous studies have suggested that Asian populations may develop metabolic risks such as type 2 diabetes and CVD at relatively lower BMI levels than Western populations [18]. In the present study, among all participants, Inactive with OW in addition to those with OB showed a significantly higher OR for incurring high ME. Thus, our results may highlight the importance of promoting PA not only among individuals with OB but also among those with OW in the Japanese population, where metabolic risks may occur at relatively lower BMI levels.

This study may provide evidence and practical considerations for implementing SHG in ways that align with broader public health goals, particularly the prevention of high ME. In men and in younger individuals, participation in either dPA or Ex was associated with lower odds of short-term high ME among individuals with OW or OB. These findings support the promotion of PA, particularly among men and younger individuals, within the framework of SHG, such as targeted PA counseling for men with OW/OB under 65. Among women with OB, those with Ex had higher ORs, which became more evident with higher thresholds for defining high ME. These findings suggest that women with OB may benefit from initiating daily PA before adding exercise to prevent high ME, highlighting the importance of gradual PA progression for women with OB. In older participants with OB, an activity level equivalent to daily PA might be appropriate. The specific reasons why initiating daily PA may be more advisable for women and older individuals with OB were not clearly identified in this study because the diagnoses at the time of healthcare utilization were not collected. However, one possible explanation for these findings may relate to differences in mobility limitations associated with OB. Previous studies on functional mobility have reported a significant impact of higher BMI on mobility-related limitations, particularly in women. A previous study showed that OB, especially with BMI levels above 35 kg/m^2^, adversely affects weight-bearing tasks such as walking, stair climbing, and chair rising ability [19]. Women are more susceptible to mobility limitations than men, with walking disability being notably prevalent [19]. In the elderly population, advancing age and fat accumulation contribute to the development of musculoskeletal degenerative diseases and sarcopenic OB. The risk of mobility disability and functional decline in older individuals increases with OB severity [20], underscoring the importance of functional capacity assessment before exercise promotion in older individuals. Accordingly, the findings from previous and present studies suggest the need for tailored PA interventions that address OB-related mobility limitations in women and older adults with the aim of preventing high ME. Nevertheless, compared to men and younger individuals, the smaller sample size of women and older individuals may limit the generalizability of the findings in our study. These findings should be interpreted as exploratory and hypothesis-generating, as the study design does not allow causal inference.

In this study, older Active participants with OB showed a higher OR for HME2009 than other groups; however, this trend disappeared when the threshold of high ME was set at 1 million yen. These individuals may have health conditions that require moderate ME but not extremely high costs, and may be active to improve their health, as individuals with emerging health problems often increase PA in response to medical advice, which could partially explain the observed associations. Because the short follow-up period may be associated with the possibility of reverse causation, these results should be interpreted with caution. Future studies on the associations between OB, PA, and ME should link ME with individual-level medical diagnoses and include longer follow-up periods to allow for the analyses of specific diseases and better address reverse causation.

AO also suggests that the association with short-term high ME may vary according to sex, age, and PA. While WC relies on a single cutoff per sex, BMI provides multiple internationally recognized thresholds, allowing for a more detailed OB classification. Our study indicated that the OR of the Ex with OB group was relatively high in women; therefore, it might be advisable to incorporate BMI alongside WC in health guidance to better tailor PA recommendations.

Although the sequential effects of individual covariates were not presented, hypertension and diabetes in Model 1 appeared to influence the estimates in the OW and OB groups without altering statistical significance. In Model 2, while the overall pattern of ORs remained largely unchanged, further adjustment for baseline ME attenuated several associations—primarily in the OW and OB groups—and these associations were no longer statistically significant across some PA categories; these findings suggest that baseline ME may warrant consideration when examining the association between PA and high ME among individuals with OW or OB.

When a higher threshold was applied to define HME, the tendency for a higher OR of Inactive with OB remained consistent across all participants and subgroups, suggesting that engaging in some form of PA may be associated with a lower occurrence of top 5% high ME or more among individuals with OB. In women, as previously described, the OR of Ex with OB was higher and statistically significant when HME2009_3 was used as the outcome, compared with the OR obtained for HME2009, which may provide insight into how PA could be initiated among women with OB. In older individuals, high OR of Active with OB was not observed when HME2009_3 was used as the outcome, indicating that reverse causation may have affected the results for HME2009. Moreover, under the higher ME threshold, some ORs that were significant for HME2009 lost statistical significance. One possible explanation for these findings is that applying a higher threshold for HME reduced the number of events. Future studies with larger sample sizes, longer follow-up periods, and methodological refinements—such as identifying all incident HME events during the follow-up period rather than assessing incidence at only two time points—may help clarify these associations.

This study had several limitations. First, the follow-up period was short; therefore, reverse causation could not be ruled out. Furthermore, OB and PA are typically associated with high-cost conditions (e.g., CVD events, complications of diabetes, and cancer) over years or decades; nevertheless, given the short follow-up period, the outcomes may have captured mainly acute events or the exacerbation of pre-existing conditions. Second, the specific diagnoses associated with ME were not identified. Third, the absolute ME values could not be examined. Fourth, the study population included only insured individuals from health insurance providers participating in a research project funded by the Ministry of Health, Labour and Welfare, potentially limiting generalizability. Fifth, although confounders were adjusted for, the possibility of residual confounding remains, such as socioeconomic status, occupational category, and unmeasured baseline comorbidities. Sixth, we could not exclude participants with diseases at baseline for which medical history was not asked in the SHG questionnaire (e.g., cancer). Thus, baseline inactivity may have reflected underlying difficulties in engaging in PA, representing one of several potential aspects of reverse causation that might have influenced our findings. Seventh, data on PA during follow-up were not available for all participants; therefore, we could not confirm whether PA status remained stable throughout the follow-up period. Finally, PA was assessed using two questions, each with yes/no responses. Such a simplified measure may not fully capture differences in intensity, frequency, or duration of activity, and might introduce misclassification bias. Although the questions used in this study may have resulted in non-differential misclassification and attenuation of the observed associations toward the null, future studies would benefit from evaluating PA using objective measures, such as accelerometer-based assessments.

## 5. Conclusions

In Japanese individuals, most of whom were employees, the present study showed that daily PA and/or Ex were associated with lower short-term high ME in men and younger individuals with OW or OB. This study also suggests that for individuals with OB, initiating daily PA and subsequently incorporating exercise might be more beneficial for women, while daily PA may be preferable for older individuals. Strategies for PA guidance in OW and OB could be tailored individually based on BMI and PA assessment using the SHC questionnaire in relation to short-term high ME. However, as the findings—particularly those in women and older adults—may be influenced by reverse causation or related factors, these observations should be confirmed in future studies, including prospective interventional studies.

## Figures and Tables

**Table 1:** Distribution of medical expenditures in 2009 among the participants.

Percentile	Corresponding Medical Expenditures (Japanese Yen)
10.0	0
20.0	0
30.0	9030
40.0	19,290
50.0	34,550
60.0	57,840
70.0	93,340
80.0	145,040
90.0	231,870
95.0	336,380
98.7	1,000,000
Maximum	20,361,990

**Table 2:** Baseline characteristics in men.

	Inactive			dPA		
	Normal Weight	Over- Weight	Obesity	Normal Weight	Over- Weight	Obesity
*n*	62,767	27,867	3927	18,606	6424	859
Age (years) †	51.9 ± 7.3	51.6 ± 7.2	49.0 ± 6.7	52.0 ± 7.6	51.5 ± 7.5	48.9 ± 6.8
BMI (kg/m^2^) †	22.3 ± 1.7	26.8 ± 1.3	32.2 ± 2.4	22.1 ± 1.7	26.7 ± 1.3	32.2 ± 2.5
Waist circumference (cm) †	81.3 ± 5.9	91.7 ± 5.2	104.1 ± 7.1	80.2 ± 5.8	91.0 ± 5.3	103.5 ± 7.1
Medication for hypertension (%)	10.3	18.1	26.5	9.5	17.9	27.2
Hypertension (%)	26.9	41.4	56.4	25.4	40.3	56.7
Medication for diabetes (%)	2.7	4.6	8.7	2.5	4.2	7.9
Diabetes (%)	7.5	12.7	22.0	6.5	12.1	21.3
Medication for dyslipidemia (%)	3.6	7.0	9.9	3.4	5.9	7.0
Dyslipidemia (%)	34.0	46.8	51.8	31.4	44.3	49.1
Current smoking (%)	54.9	50.7	52.3	55.1	48.9	52.7
Current alcohol drinking (%)						
Everyday	48.4	40.1	28.1	49.4	40.5	28.1
Sometimes	25.9	30.4	33.8	25.3	29.5	35.6
None or seldom	25.7	29.6	38.1	25.3	30.0	36.3
Medical expenditures in 2008 (yen) ‡	25,555	37,914	56,260	21,865	34,130	54,110
	Ex			Active		
	Normal weight	Over- weight	Obesity	Normal weight	Over- weight	Obesity
*n*	8951	3918	450	12,473	4584	533
Age (years) †	53.3 ± 7.6	53.0 ± 7.6	50.4 ± 7.3	53.9 ± 7.9	53.7 ± 8.0	49.5 ± 7.5
BMI (kg/m^2^) †	22.5 ± 1.6	26.7 ± 1.3	32.0 ± 2.1	22.3 ± 1.6	26.6 ± 1.3	32.1 ± 2.3
Waist circumference (cm) †	81.0 ± 5.6	90.9 ± 5.3	102.8 ± 6.9	80.2 ± 5.7	90.2 ± 5.1	103.1 ± 7.0
Medication for hypertension (%)	13.0	21.6	26.4	11.9	20.1	27.6
Hypertension (%)	28.5	42.7	56.2	29.0	43.7	55.2
Medication for diabetes (%)	4.3	6.3	9.8	4.0	6.0	10.5
Diabetes (%)	9.3	13.6	22.0	9.4	14.3	22.3
Medication for dyslipidemia (%)	4.4	7.3	8.0	3.9	7.2	10.5
Dyslipidemia (%)	31.0	42.2	45.1	29.6	41.7	46.5
Current smoking (%)	41.9	41.2	43.3	43.2	40.8	44.7
Current alcohol drinking (%)						
Everyday	46.4	41.7	27.3	47.3	41.0	27.4
Sometimes	30.8	33.8	38.4	29.5	32.4	37.9
None or seldom	22.7	24.5	34.2	23.2	26.6	34.7
Medical expenditures in 2008 (yen) ‡	36,920	54,960	63,445	31,240	45,510	66,720

†: Mean ± standard deviation, ‡: Median. BMI: body mass index. Hypertension: systolic blood pressure ≥ 140 mmHg and/or diastolic blood pressure ≥ 90 mmHg and/or medication for hypertension. Diabetes: fasting blood glucose ≥ 126 mg/dL or random blood glucose ≥ 200 mg/dL and/or medication for diabetes. Dyslipidemia: low-density lipoprotein cholesterol ≥ 140 mg/dL and/or medication for dyslipidemia.

**Table 3:** Baseline characteristics in women.

	Inactive			dPA		
	Normal-Weight	Over- Weight	Obesity	Normal-Weight	Over- Weight	Obesity
*n*	45,524	9451	2105	16,297	3059	577
Age (years) †	51.6 ± 7.1	52.8 ± 7.1	50.9 ± 7.1	51.8 ± 7.2	52.4 ± 7.3	50.4 ± 7.2
BMI (kg/m^2^) †	21.4 ± 1.7	26.8 ± 1.3	32.5 ± 2.5	21.4 ± 1.7	26.8 ± 1.3	32.6 ± 2.5
Waist circumference (cm) †	77.0 ± 6.8	89.3 ± 6.4	100.4 ± 8.3	76.6 ± 6.8	88.9 ± 6.4	100.4 ± 8.1
Medication for hypertension (%)	7.6	18.2	26.8	7.7	16.7	23.2
Hypertension (%)	17.6	37.9	54.9	17.8	36.7	50.6
Medication for diabetes (%)	0.8	2.9	5.1	0.7	2.1	6.6
Diabetes (%)	2.0	6.8	12.7	1.8	6.0	15.8
Medication for dyslipidemia (%)	5.2	9.7	11.0	4.8	8.7	11.3
Dyslipidemia (%)	34.1	51.2	54.8	32.2	49.9	50.4
Current smoking (%)	16.8	16.4	17.8	17.5	16.5	19.9
Current alcohol drinking (%)						
Everyday	13.7	9.9	6.5	14.0	9.3	6.2
Sometimes	28.6	27.2	25.8	28.6	27.2	29.3
None or seldom	57.7	63.6	67.7	57.4	63.6	64.5
Medical expenditures in baseline (yen) ‡	42,436	56,900	73,513	40,377	52,160	66,700
	Ex			Active		
	Normal-weight	Over- weight	Obesity	Normal-weight	Over- weight	Obesity
*n*	5263	982	173	8486	1550	218
Age (years) †	53.8 ± 7.4	54.8 ± 7.0	53.4 ± 7.1	54.5 ± 7.5	55.5 ± 7.5	52.1 ± 7.6
BMI (kg/m^2^) †	21.5 ± 1.7	26.7 ± 1.3	32.1 ± 2.4	21.5 ± 1.7	26.7 ± 1.3	32.5 ± 2.5
Waist circumference (cm) †	77.1 ± 6.9	89.3 ± 6.5	100.0 ± 7.5	77.0 ± 6.9	88.8 ± 6.5	100.0 ± 8.1
Medication for hypertension (%)	8.8	22.2	36.4	9.4	19.2	25.7
Hypertension (%)	18.9	42.1	59.0	20.8	39.9	55.0
Medication for diabetes (%)	1.2	4.8	9.2	1.3	4.1	8.3
Diabetes (%)	2.5	9.7	16.2	2.5	8.6	17.0
Medication for dyslipidemia (%)	7.1	12.9	16.2	7.6	11.0	14.2
Dyslipidemia (%)	37.3	55.7	55.5	36.5	54.8	56.0
Current smoking (%)	12.3	12.8	12.7	13.9	11.8	11.9
Current alcohol drinking (%)						
Everyday	14.9	11.3	6.4	15.0	8.5	4.6
Sometimes	31.9	30.7	19.7	29.9	25.7	22.9
None or seldom	53.2	58.0	74.0	55.1	65.7	72.5
Medical expenditures in baseline (yen) ‡	48,960	80,015	91,010	49,540	69,225	80,825

†: Mean ± standard deviation, ‡: Median. BMI: body mass index. Hypertension: systolic blood pressure ≥ 140 mmHg and/or diastolic blood pressure ≥ 90 mmHg and/or medication for hypertension. Diabetes: fasting blood glucose ≥ 126 mg/dL or random blood glucose ≥ 200 mg/dL and/or medication for diabetes. Dyslipidemia: low-density lipoprotein cholesterol ≥ 140 mg/dL and/or medication for dyslipidemia.

**Table 4:** ORs of the BMI categories and the combination of PA and BMI categories for HME2009 in all participants.

	Normal Weight		Overweight		Obesity	
**Comparison among BMI categories**						
*n*	178,367		57,835		8842	
HME2009 (%)	4.6		6.1		8.0	
NoME2009 (%)	22.9		21.2		19.3	
Sex and age-adjusted ORs	1.00	(Ref.)	1.33	(1.28–1.39)	2.09	(1.93–2.27)
Multivariable-adjusted ORs 1 ^a^	1.00	(Ref.)	1.16	(1.11–1.21)	1.52	(1.39–1.65)
Multivariable-adjusted ORs 2 ^b^	1.00	(Ref.)	1.08	(1.04–1.13)	1.25	(1.15–1.36)
Predictive probability (%)	3		4		4	
**Comparison among 12 groups stratified by physical activity and BMI categories**						
**Inactive**						
*n*	108,291		37,318		6032	
HME2009 (%)	4.6		6.0		8.1	
NoME2009 (%)	22.8		21.4		19.5	
Sex and age-adjusted ORs	1.00	(Ref.)	1.31	(1.25–1.38)	2.10	(1.91–2.32)
Multivariable-adjusted ORs 1 ^a^	1.00	(Ref.)	1.14	(1.08–1.20)	1.53	(1.38–1.69)
Multivariable-adjusted ORs 2 ^b^	1.00	(Ref.)	1.07	(1.01–1.13)	1.26	(1.14–1.40)
Predictive probability (%)	3		4		4	
**dPA**						
*n*	34,903		9483		1436	
HME2009 (%)	4.1		5.5		7.0	
NoME2009 (%)	24.3		21.7		19.4	
Sex and age-adjusted ORs	0.89	(0.84–0.94)	1.20	(1.10–1.32)	1.82	(1.48–2.23)
Multivariable-adjusted Ors 1 ^a^	0.90	(0.85–0.96)	1.06	(0.97–1.17)	1.31	(1.07–1.62)
Multivariable-adjusted ORs 2 ^b^	0.93	(0.87–0.99)	1.03	(0.93–1.13)	1.09	(0.88–1.35)
Predictive probability (%)	3		4		4	
**Ex**						
*n*	14,214		4900		623	
HME2009 (%)	5.1		6.5		8.7	
NoME2009 (%)	20.4		19.0		17.7	
Sex and age-adjusted ORs	1.00	(0.93–1.09)	1.31	(1.17–1.47)	2.04	(1.54–2.70)
Multivariable-adjusted ORs 1 ^a^	1.00	(0.92–1.09)	1.14	(1.01–1.29)	1.49	(1.12–1.98)
Multivariable-adjusted ORs 2 ^b^	0.97	(0.89–1.06)	0.99	(0.88–1.12)	1.21	(0.90–1.63)
Predictive probability (%)	3		3		4	
**Active**						
*n*	20,959		6134		751	
HME2009 (%)	5.0		6.9		7.9	
NoME2009 (%)	22.8		21.2		19.2	
Sex and age-adjusted ORs	0.95	(0.88–1.01)	1.33	(1.19–1.47)	1.93	(1.47–2.52)
Multivariable-adjusted ORs 1 ^a^	0.95	(0.88–1.01)	1.16	(1.05–1.29)	1.39	(1.06–1.83)
Multivariable-adjusted ORs 2 ^b^	0.95	(0.88–1.02)	1.08	(0.97–1.20)	1.11	(0.83–1.47)
Predictive probability (%)	3		4		4	

HME2009, high medical expenditures in next year (2009); NoME2009, no medical expenditures in next year (2009); ORs, odds ratio; BMI, body mass index. Values in parentheses following the odds ratios indicate 95% confidence intervals. ^a^, Adjusted for age, hypertension, diabetes mellitus, dyslipidemia (LDL-C and medication), current smoking and current alcohol consumption status (Model 1). ^b^, Further adjusted for medical expenditures in the baseline year in addition to Model 1 (Model 2).

**Table 5:** ORs of the BMI categories and the combination of PA and BMI categories for HME2009 by sex.

	Normal Weight		Overweight		Obesity	
**Men**						
**Comparison among BMI categories**						
*n*	102,797		42,793		5769	
HME2009 (%)	4.7		6.0		8.0	
NoME2009 (%)	27.3		23.0		21.2	
Sex and age-adjusted ORs	1.00	(Ref.)	1.33	(1.26–1.40)	2.21	(2.00–2.44)
Multivariable-adjusted ORs 1 ^a^	1.00	(Ref.)	1.15	(1.10–1.21)	1.57	(1.42–1.75)
Multivariable-adjusted ORs 2 ^b^	1.00	(Ref.)	1.07	(1.01–1.12)	1.26	(1.13–1.41)
Predictive probability (%)	3		4		4	
**Comparison among 12 groups stratified by physical activity and BMI categories**						
**Inactive**						
*n*	62,767		27,867		3927	
HME2009 (%)	4.7		6.0		8.1	
NoME2009 (%)	27.2		23.1		21.8	
Sex and age-adjusted ORs	1.00	(Ref.)	1.34	(1.25–1.42)	2.23	(1.97–2.52)
Multivariable-adjusted ORs 1 ^a^	1.00	(Ref.)	1.16	(1.09–1.23)	1.58	(1.40–1.80)
Multivariable-adjusted ORs 2 ^b^	1.00	(Ref.)	1.08	(1.01–1.16)	1.28	(1.12–1.46)
Predictive probability (%)	4		4		4	
**dPA**						
*n*	18,606		6424		859	
HME2009 (%)	4.4		5.3		7.2	
NoME2009 (%)	30.1		24.3		20.4	
Sex and age-adjusted ORs	0.92	(0.85–1.00)	1.17	(1.04–1.31)	1.97	(1.51–2.56)
Multivariable-adjusted ORs 1 ^a^	0.95	(0.87–1.03)	1.03	(0.92–1.16)	1.42	(1.08–1.85)
Multivariable-adjusted ORs 2 ^b^	0.98	(0.90–1.06)	0.98	(0.86–1.10)	1.16	(0.88–1.53)
Predictive probability (%)	3		3		4	
**Ex**						
*n*	8951		3918		450	
HME2009 (%)	5.2		6.5		8.2	
NoME2009 (%)	23.3		19.9		19.6	
Sex and age-adjusted ORs	1.02	(0.93–1.13)	1.32	(1.16–1.51)	2.04	(1.45–2.87)
Multivariable-adjusted ORs 1 ^a^	1.01	(0.91–1.12)	1.15	(1.01–1.32)	1.49	(1.05–2.10)
Multivariable-adjusted ORs 2 ^b^	0.97	(0.87–1.08)	1.00	(0.87–1.15)	1.17	(0.82–1.68)
Predictive probability (%)	3		4		4	
**Active**						
*n*	12,473		4584		533	
HME2009 (%)	5.2		6.8		8.6	
NoME2009 (%)	26.5		23.0		19.3	
Sex and age-adjusted ORs	0.97	(0.89–1.06)	1.32	(1.16–1.51)	2.27	(1.67–3.08)
Multivariable-adjusted ORs 1 ^a^	0.96	(0.88–1.05)	1.15	(1.01–1.30)	1.61	(1.18–2.20)
Multivariable-adjusted ORs 2 ^b^	0.96	(0.88–1.06)	1.05	(0.92–1.19)	1.24	(0.89–1.71)
Predictive probability (%)	3		4		4	
**Women**						
**Comparison among BMI categories**						
*n*	75,570		15,042		3073	
HME2009 (%)	4.4		6.2		7.8	
NoME2009 (%)	16.9		16.1		15.8	
Sex and age-adjusted ORs ^a^	1.00	(Ref.)	1.38	(1.28–1.48)	1.96	(1.71–2.24)
Multivariable-adjusted ORs 1	1.00	(Ref.)	1.19	(1.10–1.29)	1.44	(1.25–1.66)
Multivariable-adjusted ORs 2 ^b^	1.00	(Ref.)	1.13	(1.04–1.23)	1.25	(1.07–1.45)
Predictive probability (%)	3		4		4	
**Comparison among 12 groups stratified by physical activity and BMI categories**						
**Inactive**						
*n*	45,524		9451		2105	
HME2009 (%)	4.5		6.0		8.1	
NoME2009 (%)	16.6		16.2		15.1	
Sex and age-adjusted ORs	1.00	(Ref.)	1.30	(1.18–1.43)	1.97	(1.67–2.31)
Multivariable-adjusted ORs 1 ^a^	1.00	(Ref.)	1.13	(1.02–1.25)	1.47	(1.24–1.74)
Multivariable-adjusted ORs 2 ^b^	1.00	(Ref.)	1.07	(0.96–1.18)	1.28	(1.07–1.52)
Predictive probability (%)	3		3		4	
**dPA**						
*n*	16,297		3059		577	
HME2009 (%)	3.8		5.9		6.8	
NoME2009 (%)	17.7		16.4		18.0	
Sex and age-adjusted ORs	0.84	(0.77–0.92)	1.30	(1.11–1.52)	1.64	(1.18–2.28)
Multivariable-adjusted ORs 1 ^a^	0.85	(0.77–0.93)	1.15	(0.98–1.35)	1.19	(0.85–1.66)
Multivariable-adjusted ORs 2 ^b^	0.87	(0.79–0.96)	1.16	(0.98–1.37)	1.00	(0.70–1.42)
Predictive probability (%)	3		4		3	
**Ex**						
*n*	5263		982		173	
HME2009 (%)	4.8		6.6		9.8	
NoME2009 (%)	15.5		15.4		12.7	
Sex and age-adjusted ORs	0.98	(0.86–1.12)	1.32	(1.02–1.70)	2.16	(1.30–3.58)
Multivariable-adjusted ORs 1 ^a^	1.00	(0.88–1.15)	1.12	(0.86–1.45)	1.55	(0.93–2.59)
Multivariable-adjusted ORs 2 ^b^	0.99	(0.86–1.14)	0.99	(0.76–1.30)	1.40	(0.82–2.39)
Predictive probability (%)	3		3		4	
**Active**						
*n*	8486		1550		218	
HME2009 (%)	4.7		7.2		6.0	
NoME2009 (%)	17.5		16.1		18.8	
Sex and age-adjusted ORs	0.92	(0.82–1.03)	1.39	(1.14–1.70)	1.32	(0.75–2.33)
Multivariable-adjusted ORs 1 ^a^	0.93	(0.83–1.04)	1.22	(1.00–1.49)	0.97	(0.55–1.71)
Multivariable-adjusted ORs 2 ^b^	0.93	(0.82–1.04)	1.19	(0.96–1.47)	0.87	(0.48–1.56)
Predictive probability (%)	3		4		3	

HME2009, high medical expenditures in next year (2009); NoME2009, no medical expenditures in next year (2009); ORs, odds ratio; BMI, body mass index. Values in parentheses following the odds ratios indicate 95% confidence intervals. ^a^, Adjusted for age, hypertension, diabetes mellitus, dyslipidemia (LDL-C and medication), current smoking and current alcohol consumption status (Model 1). ^b^, Further adjusted for medical expenditures in the baseline year in addition to Model 1 (Model 2).

**Table 6:** ORs of the BMI categories and the combination of PA and BMI categories for HME2009 by age groups.

	Normal Weight		Overweight		Obesity	
**<65 years**						
**Comparison among BMI categories**						
*n*	170,143		55,082		8618	
HME2009 (%)	4.3		5.8		7.8	
NoME2009 (%)	23.1		21.4		19.5	
Sex and age-adjusted ORs	1.00	(Ref.)	1.34	(1.28–1.40)	2.11	(1.94–2.29)
Multivariable-adjusted ORs 1 ^a^	1.00	(Ref.)	1.16	(1.11–1.21)	1.52	(1.39–1.65)
Multivariable-adjusted ORs 2 ^b^	1.00	(Ref.)	1.08	(1.03–1.13)	1.25	(1.14–1.36)
Predictive probability (%)	3		3		4	
**Comparison among 12 groups stratified by physical activity and BMI categories**						
**Inactive**						
*n*	104,468		35,930		5904	
HME2009 (%)	4.4		5.8		7.9	
NoME2009 (%)	23.0		21.5		19.6	
Sex and age-adjusted ORs	1.00	(Ref.)	1.32	(1.25–1.39)	2.11	(1.91–2.33)
Multivariable-adjusted ORs 1 ^a^	1.00	(Ref.)	1.14	(1.08–1.21)	1.52	(1.37–1.68)
Multivariable-adjusted ORs 2 ^b^	1.00	(Ref.)	1.07	(1.01–1.13)	1.26	(1.13–1.40)
Predictive probability (%)	3		4		4	
**dPA**						
*n*	33,348		9068		1405	
HME2009 (%)	3.9		5.3		7.1	
NoME2009 (%)	24.4		21.9		19.5	
Sex and age-adjusted ORs	0.89	(0.84–0.95)	1.21	(1.10–1.34)	1.90	(1.55–2.34)
Multivariable-adjusted ORs 1 ^a^	0.90	(0.85–0.96)	1.06	(0.96–1.17)	1.37	(1.11–1.69)
Multivariable-adjusted ORs 2 ^b^	0.93	(0.87–0.99)	1.02	(0.93–1.13)	1.12	(0.90–1.40)
Predictive probability (%)	3		3		4	
**Ex**						
*n*	13,251		4564		595	
HME2009 (%)	4.8		6.0		8.4	
NoME2009 (%)	20.5		19.1		18.0	
Sex and age-adjusted ORs	1.01	(0.93–1.10)	1.30	(1.14–1.47)	2.08	(1.55–2.79)
Multivariable-adjusted ORs 1 ^a^	1.00	(0.92–1.09)	1.13	(0.99–1.28)	1.51	(1.12–2.03)
Multivariable-adjusted ORs 2 ^b^	0.97	(0.89–1.06)	0.98	(0.86–1.12)	1.25	(0.91–1.70)
Predictive probability (%)	3		3		4	
**Active**						
*n*	19,076		5520		714	
HME2009 (%)	4.6		6.4		7.3	
NoME2009 (%)	23.3		21.8		19.6	
Sex and age-adjusted ORs	0.94	(0.88–1.02)	1.35	(1.21–1.52)	1.90	(1.43–2.53)
Multivariable-adjusted ORs 1 ^a^	0.94	(0.87–1.02)	1.17	(1.04–1.31)	1.35	(1.01–1.80)
Multivariable-adjusted ORs 2 ^b^	0.94	(0.87–1.02)	1.08	(0.96–1.22)	1.05	(0.78–1.42)
Predictive probability (%)	3		4		3	
**≥65 years**						
**Comparison among BMI categories**						
*n*	8224		2753		224	
HME2009 (%)	9.4		11.9		15.6	
NoME2009 (%)	19.2		16.9		12.5	
Sex and age-adjusted ORs	1.00	(Ref.)	1.28	(1.12–1.47)	1.77	(1.22–2.56)
Multivariable-adjusted ORs 1 ^a^	1.00	(Ref.)	1.16	(1.01–1.33)	1.40	(0.96–2.03)
Multivariable-adjusted ORs 2 ^b^	1.00	(Ref.)	1.12	(0.96–1.29)	1.12	(0.76–1.66)
Predictive probability (%)	7		8		8	
**Comparison among 12 groups stratified by physical activity and BMI categories**						
**Inactive**						
*n*	3823		1388		128	
HME2009 (%)	9.8		12.0		18.0	
NoME2009 (%)	18.2		17.1		12.5	
Sex and age-adjusted ORs	1.00	(Ref.)	1.24	(1.02–1.51)	1.99	(1.25–3.17)
Multivariable-adjusted ORs 1 ^a^	1.00	(Ref.)	1.12	(0.92–1.37)	1.62	(1.01–2.59)
Multivariable-adjusted ORs 2 ^b^	1.00	(Ref.)	1.12	(0.91–1.37)	1.20	(0.73–1.97)
Predictive probability (%)	8		8		9	
**dPA**						
*n*	1555		415		31	
HME2009 (%)	8.6		10.8		3.2	
NoME2009 (%)	22.2		17.3		16.1	
Sex and age-adjusted ORs	0.86	(0.70–1.06)	1.10	(0.79–1.52)	0.28	(0.04–2.05)
Multivariable-adjusted ORs 1 ^a^	0.88	(0.72–1.09)	1.04	(0.75–1.44)	0.21	(0.03–1.55)
Multivariable-adjusted ORs 2 ^b^	0.92	(0.74–1.15)	1.02	(0.72–1.44)	0.22	(0.03–1.65)
Predictive probability (%)	7		8		2	
**Ex**						
*n*	963		336		28	
HME2009 (%)	9.2		13.4		14.3	
NoME2009 (%)	19.6		16.7		10.7	
Sex and age-adjusted ORs	0.94	(0.73–1.19)	1.40	(1.00–1.95)	1.62	(0.56–4.70)
Multivariable-adjusted ORs 1 ^a^	0.96	(0.75–1.23)	1.24	(0.89–1.74)	1.22	(0.42–3.57)
Multivariable-adjusted ORs 2 ^b^	0.97	(0.75–1.25)	1.10	(0.77–1.57)	0.89	(0.29–2.73)
Predictive probability (%)	7		8		7	
**Active**						
*n*	1883		614		37	
HME2009 (%)	9.2		11.4		18.9	
NoME2009 (%)	18.6		16.3		10.8	
Sex and age-adjusted ORs	0.93	(0.77–1.12)	1.15	(0.88–1.51)	2.14	(0.93–4.92
Multivariable-adjusted ORs 1 ^a^	0.95	(0.78–1.15)	1.08	(0.82–1.42)	1.79	(0.77–4.13)
Multivariable-adjusted ORs 2 ^b^	0.97	(0.80–1.19)	1.06	(0.80–1.42)	1.83	(0.76–4.42)
Predictive probability (%)	7		8		13	

HME2009, high medical expenditures in next year (2009); NoME2009, no medical expenditures in next year (2009); ORs, odds ratio; BMI, body mass index. Values in parentheses following the odds ratios indicate 95% confidence intervals. ^a^, Adjusted for age, hypertension, diabetes mellitus, dyslipidemia (LDL-C and medication), current smoking and current alcohol consumption status (Model 1). ^b^, Further adjusted for medical expenditures in the baseline year in addition to Model 1 (Model 2).

## Data Availability

The datasets presented in this article are not readily available due to ethical approval requirements and the terms of consent under which the data were obtained from the data provider. Requests to access the datasets should be directed to the corresponding author.

## Supplementary Materials

The following supporting information can be downloaded at: https://www.mdpi.com/article/10.3390/healthcare14060777/s1. Additional File S1 (.xls): Table S1. ORs of AO and the combination of PA and AO categories for HME2009 in all participants. Table S2. ORs of the combination of PA and AO categories for HME2009 by sex. Table S3. ORs of the combination of PA and AO categories for HME2009 by age groups. Additional File S2 (.docx): Figure S1. Multivariable-adjusted ORs of the groups classified by the combination of BMI and physical activity categories for HME2009, HME2009_2 and HME2009_3. (a) All participants. (b) Men. (c) Women. (d) Younger participants (<65 years). (e) Older participants (≥65 years). ORs, odds ratio; NW, normal weight; OW, overweight; OB, obesity; HME, high medical expenditures. HME2009: 95.0–100.0 percentile of the distribution in medical expenditures in 2009 among all participants. HME2009_2: 96.0–100.0 percentile of the distribution in medical expenditures in 2009 among all participants. HME2009_3: Expenditures of Japanese yen 1,000,000 or more in 2009. The multivariable-adjusted ORs were adjusted for age, sex, hypertension, diabetes, dyslipidemia, smoking, alcohol consumption, and medical expenditures in 2008 (Model 2).

## Abbreviations

The following abbreviations are used in this manuscript:

CVD	cardiovascular disease
ME	medical expenditures
EHI	Employees’ Health Insurance
SHC	Specific Health Checkups
SHG	Specific Health Guidance
PA	physical activity
BMI	body mass index
FY	fiscal year
NHI	National Health Insurance
JPY	Japanese yen
NW	normal weight
OW	overweight
OB	obesity
AO	abdominal obesity
WC	waist circumference
SBP	systolic blood pressure
DBP	diastolic blood pressure
LDL-C	low-density lipoprotein cholesterol
dPA	daily physical activity
Ex	exercising
HME2009	high medical expenditures in 2009
noME2008	zero medical expenditures in 2008
noME2009	zero medical expenditures in 2009
ORs	odds ratios
95% CI	95% confidence intervals
ME2008	medical expenditures in 2008

## References

[1] Kong Y., Yang H., Nie R., Zhang X., Zuo F., Zhang H., Nian X. (2025). Obesity: Pathophysiology and therapeutic interventions. Mol. Biomed..

[2] Ministry of Health, Labour and Welfare Specific Health Checkups and Specific Health Guidance. https://www.mhlw.go.jp/english/wp/wp-hw3/dl/2-007.pdf?utm_source=chatgpt.com.

[3] Ministry of Health, Labour and Welfare Physical Activity Guide for Health Promotion 2023. https://www.mhlw.go.jp/stf/seisakunitsuite/bunya/kenkou_iryou/kenkou/undou/index.html.

[4] Heron L., Tully M.A., Kee F., O’Neill C. (2023). Inpatient care utilisation and expenditure associated with objective physical activity: Econometric analysis of the UK Biobank. Eur. J. Health Econ..

[5] Buder I., Waitzman N., Zick C. (2020). The medical costs of low leisure-time physical activity among working-age adults: Gender and minority status matter. Prev. Med..

[6] Lei X.-L., Gao K., Wang H., Chen W., Chen G.-R., Wen X. (2023). The role of physical activity on healthcare utilization in China. BMC Public Health.

[7] Pronk N.P., Goodman M.J., O’Connor P.J., Martinson B.C. (1999). Relationship between modifiable health risks and short-term health care charges. JAMA.

[8] Li C.-Y., Sung F.-C. (1999). A review of the healthy worker effect in occupational epidemiology. Occup. Med..

[9] Ogawa W., Hirota Y., Miyazaki S., Nakamura T., Ogawa Y., Shimomura I., Yamauchi T., Yokote K., on behalf of the Creation Committee for Guidelines for the Management of Obesity Disease 2022 by Japan Society for the Study of Obesity (JASSO) (2024). Definition, criteria, and core concepts of guidelines for the management of obesity disease in Japan. Endocr. J..

[10] Okayama A. Final Report of a Health, Labour and Welfare Sciences Research Grant–Funded Project: The Influence of Specific Health Checkups and Specific Health Guidance by Medical Insurers on Medical Expenses Ministry of Health, Labour, and Welfare Grants System. https://mhlw-grants.niph.go.jp/project/17625?utm.

[11] Rattanavipapong W., Wang Y., Butchon R., Kittiratchakool N., Thammatacharee J., Teerawattananon Y., Isaranuwatchai W. (2021). Retrospective secondary data analysis to identify high-cost users in inpatient department of hospitals in Thailand, a middle-income country with universal healthcare coverage. BMJ Open.

[12] Okamura T., Tsukamoto K., Arai H., Fujioka Y., Ishigaki Y., Koba S., Ohmura H., Shoji T., Yokote K., Yoshida H. (2022). Japan Atherosclerosis Society (JAS) guidelines for prevention of atherosclerotic cardiovascular diseases. J. Atheroscler. Thromb..

[13] NCD Risk Factor Collaboration (NCD-RisC) (2024). Worldwide trends in underweight and obesity from 1990 to 2022: A pooled analysis of 3663 population-representative studies with 222 million children, adolescents, and adults. Lancet.

[14] Ward Z.J., Bleich S.N., Long M.W., Gortmaker S.L. (2021). Association of body mass index with health care expenditures in the United States by age and sex. PLoS ONE.

[15] Dixon P., Smith G.D., Hollingworth W. (2019). The Association Between Adiposity and Inpatient Hospital Costs in the UK Biobank Cohort. Appl. Health Econ. Health Policy.

[16] Coughlan D., Saint-Maurice P.F., Carlson S.A., Fulton J., Matthews C.E. (2021). Leisure time physical activity throughout adulthood is associated with lower medicare costs: Evidence from the linked NIH-AARP diet and health study cohort. BMJ Open Sport Exerc. Med..

[17] de Morais L.C., Araujo M.Y.C., Koyama K.A.K., Lemes Í.R., Fernandes R.A., Turi-Lynch B.C., Monteiro H.L., Codogno J.S. (2025). Healthcare costs according to obesity and physical activity: 8-year longitudinal study among patients assisted by the Brazilian National Healthcare System. Obes. Res. Clin. Pract..

[18] WHO Expert Consultation (2004). Appropriate body-mass index for Asian populations and its implications for policy and intervention strategies. Lancet.

[19] Mohan D.M., Al Anouti F., Kohli N., Khalaf K. (2025). Association of obesity with musculoskeletal health and functional mobility in females—A systematic review. Int. J. Obes..

[20] Vincent H.K., Raiser S.N., Vincent K.R. (2012). The aging musculoskeletal system and obesity-related considerations with exercise. Ageing Res. Rev..

