# Supplementary material for: Obesity, Physical Activity and Occurrence of High Medical Expenditures at One-Year Follow-Up Among Japanese Beneficiaries of Employment-Based Health Insurances: An Analysis Based on a Nationwide Health Checkup Questionnaire
Source: Healthcare (Basel). 2026 Mar 19;14(6):777. doi: 10.3390/healthcare14060777 (PMC13027250; doi:10.3390/healthcare14060777)

Supplementary Figure S1. Multivariable-adjusted ORs of the groups classified by the combination of BMI and PA categories for HME2009, HME2009_2 and HME2009_3.

Suppl Fig S1a: All participants.

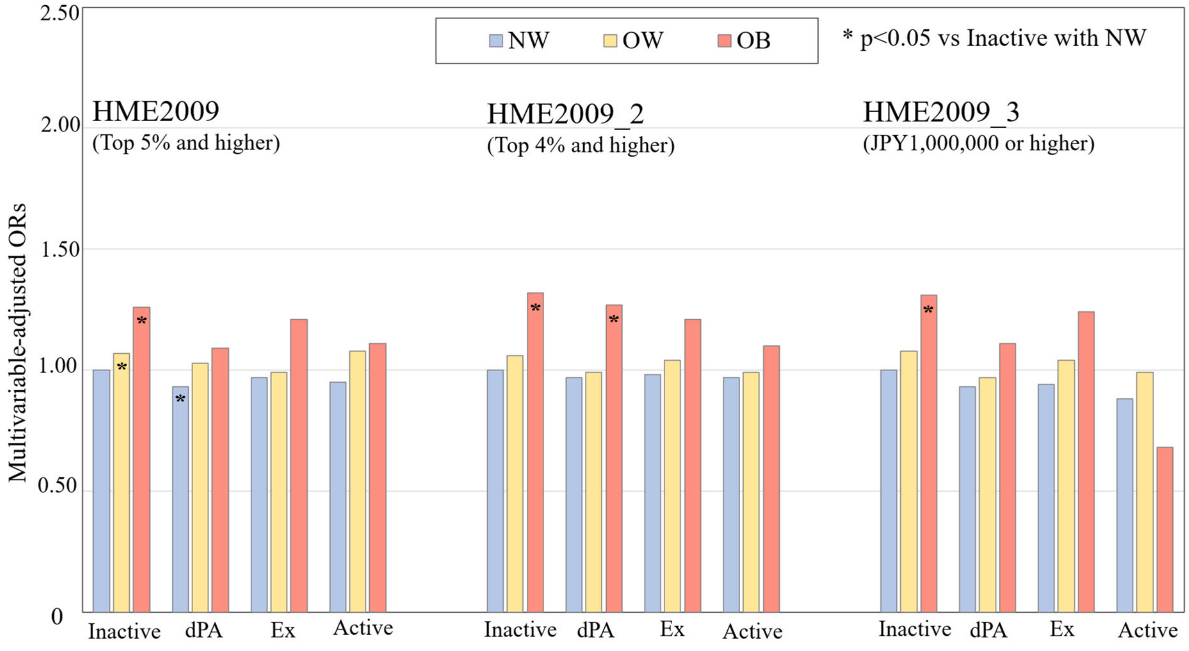

(The results of sex-and age-group-specific analyses are shown in pairs from the following pages.)

Suppl Figure S1b: Men.

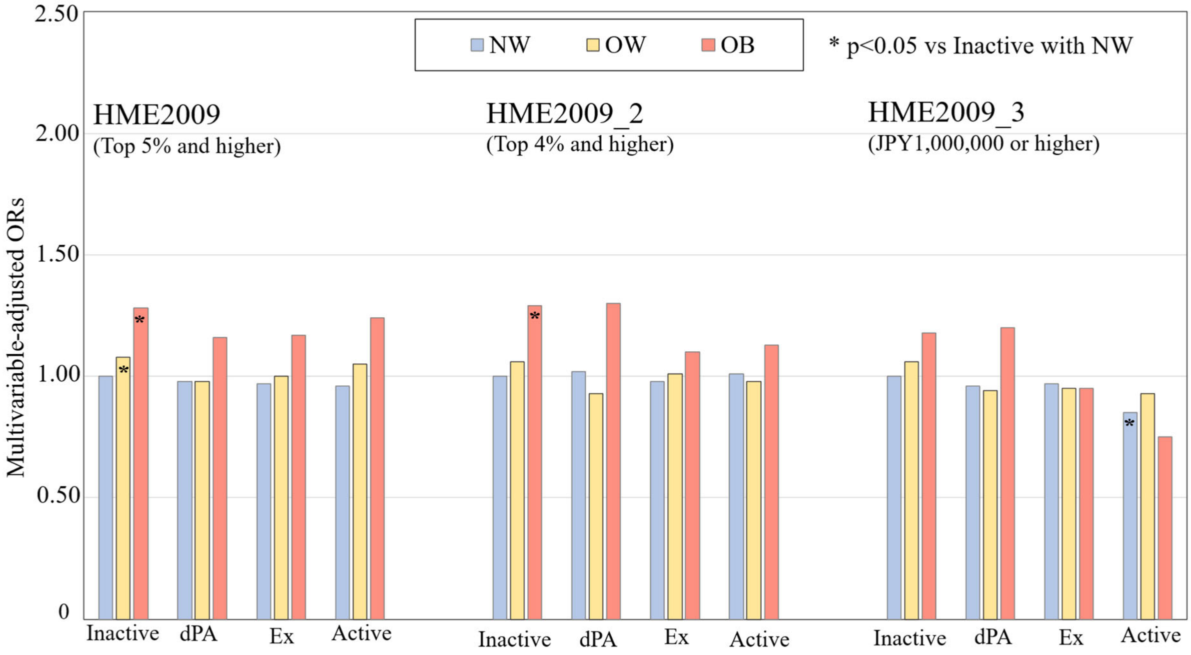

Suppl Figure S1c: Women.

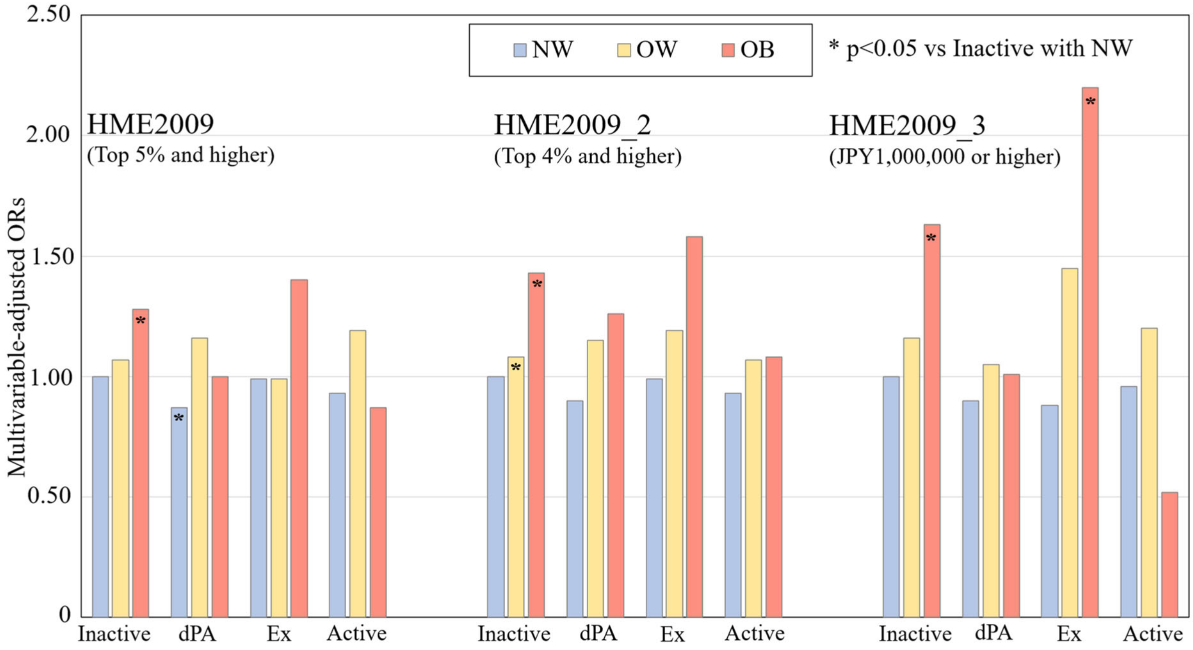

Suppl Figure S1d: Younger participants (<65 years)

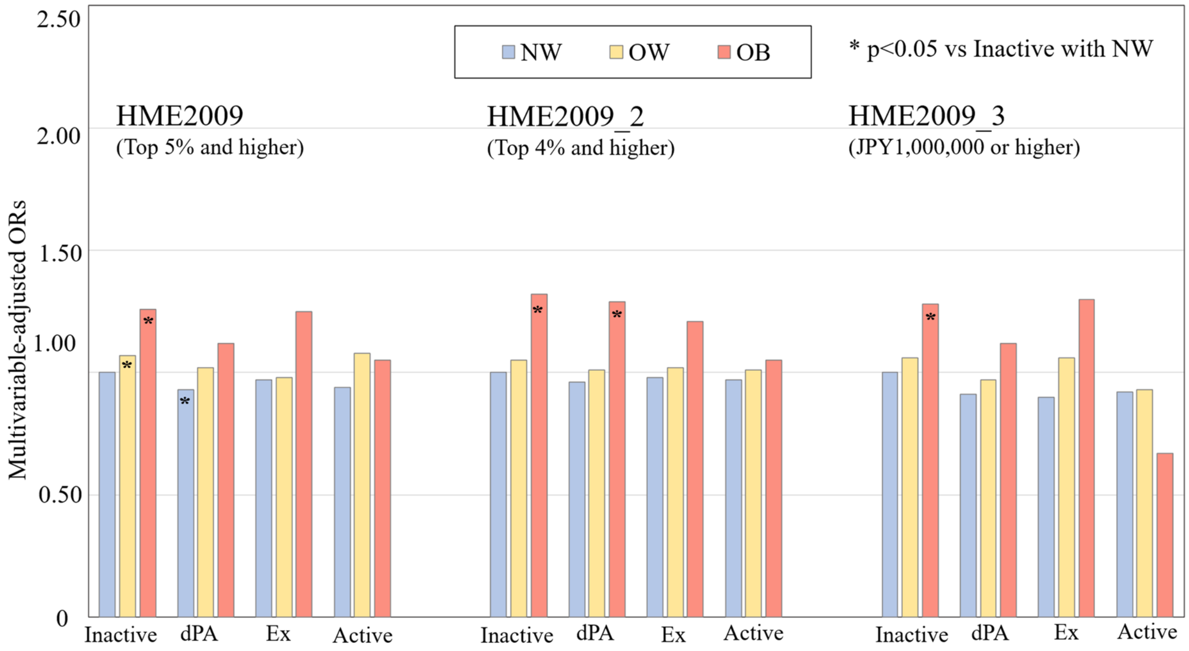

Suppl Figure S1e: Older participants (≥65 years).

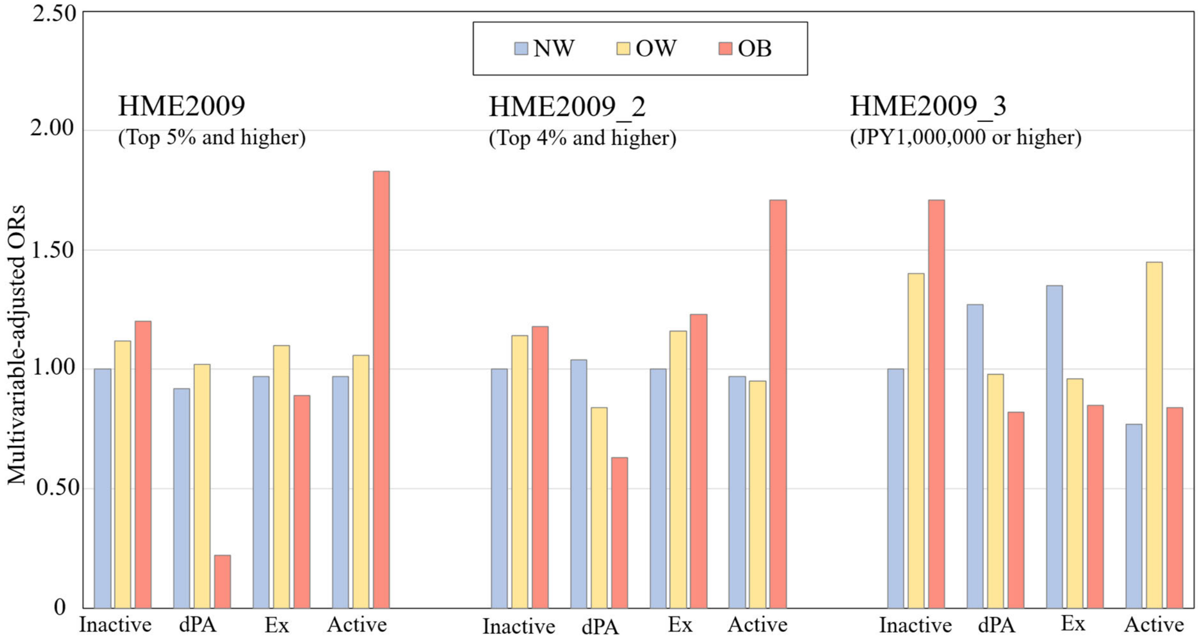

Supplement: Supplementary file 1 [file healthcare-14-00777-s001.zip › Figure S1.pdf]
